# The Netherlands Arrhythmogenic Cardiomyopathy Registry: design and status update

**DOI:** 10.1007/s12471-019-1270-1

**Published:** 2019-04-17

**Authors:** L. P. Bosman, T. E. Verstraelen, F. H. M. van Lint, M. G. P. J. Cox, J. A. Groeneweg, T. P. Mast, P. A. van der Zwaag, P. G. A. Volders, R. Evertz, L. Wong, N. M. S. de Groot, K. Zeppenfeld, J. F. van der Heijden, M. P. van den Berg, A. A. M. Wilde, F. W. Asselbergs, R. N. W. Hauer, A. S. J. M. te Riele, J. P. van Tintelen, Netherlands ACM Registry, A. F. Baas, D. Q. C. M. Barge-Schaapveld, S. M. Boekholdt, M. J. M. Cramer, D. Dooijes, J. D. H. Jongbloed, P. Loh, R. N. Planken, N. H. J. Prakken, J. J. van der Smagt, A. C. van der Wal, A. J. Teske, T. A. B. van Veen, B. K. Velthuis, A. Vink, S. C. Yap

**Affiliations:** 1 Durrer Centre for Cardiovascular Research, Netherlands Heart Institute, Utrecht, The Netherlands; 2 Department of Clinical and Experimental Cardiology, Amsterdam UMC, Heart Centre, Amsterdam, The Netherlands; 3 Department of Clinical Genetics, Amsterdam UMC, AMC, Amsterdam, The Netherlands; 4 Department of Cardiology, UMC Utrecht, University of Utrecht, Utrecht, The Netherlands; 5 Department of Genetics, UMC Groningen, Groningen, The Netherlands; 6 Department of Cardiology, Maastricht UMC, Maastricht, The Netherlands; 7 Department of Cardiology, Radboud MC, Nijmegen, The Netherlands; 8 Department of Cardiology, Amsterdam UMC, VUMC, Amsterdam, The Netherlands; 9 Department of Cardiology, Erasmus MC, Rotterdam, The Netherlands; 10 Department of Cardiology, LUMC, Leiden, The Netherlands; 11 Department of Cardiology, UMC Groningen, Groningen, The Netherlands

**Keywords:** Cardiomyopathies, Arrhythmogenic right ventricular dysplasia, Research design, Registries

## Abstract

**Background:**

Clinical research on arrhythmogenic cardiomyopathy (ACM) is typically limited by small patient numbers, retrospective study designs, and inconsistent definitions.

**Aim:**

To create a large national ACM patient cohort with a vast amount of uniformly collected high-quality data that is readily available for future research.

**Methods:**

This is a multicentre, longitudinal, observational cohort study that includes (1) patients with a definite ACM diagnosis, (2) at-risk relatives of ACM patients, and (3) ACM-associated mutation carriers. At baseline and every follow-up visit, a medical history as well information regarding (non-)invasive tests is collected (e. g. electrocardiograms, Holter recordings, imaging and electrophysiological studies, pathology reports, etc.). Outcome data include (non-)sustained ventricular and atrial arrhythmias, heart failure, and (cardiac) death. Data are collected on a research electronic data capture (REDCap) platform in which every participating centre has its own restricted data access group, thus empowering local studies while facilitating data sharing.

**Discussion:**

The Netherlands ACM Registry is a national observational cohort study of ACM patients and relatives. Prospective and retrospective data are obtained at multiple time points, enabling both cross-sectional and longitudinal research in a hypothesis-generating approach that extends beyond one specific research question. In so doing, this registry aims to (1) increase the scientific knowledge base on disease mechanisms, genetics, and novel diagnostic and treatment strategies of ACM; and (2) provide education for physicians and patients concerning ACM, e. g. through our website (www.acmregistry.nl) and patient conferences.

**Electronic supplementary material:**

The online version of this article (10.1007/s12471-019-1270-1) contains supplementary material, which is available to authorized users.

## Introduction

Arrhythmogenic cardiomyopathy (ACM), including its major subform arrhythmogenic right ventricular cardiomyopathy, is a relatively rare heart muscle disease that affects approximately 1:1,000–5,000 people [1, 2]. It is characterised by an increased risk of ventricular arrhythmias, sudden cardiac death, and progressively deteriorating ventricular function due to intercalated disk remodelling and fibro-fatty myocardial replacement [3]. ACM can present both in isolated and in familial forms and is consistent with an autosomal dominant inheritance pattern with incomplete penetrance and variable expressivity.

ACM was first described by Marcus et al. in 1982 [4]. Since then, considerable advancements have been made that have improved our knowledge of this clinical entity. Nonetheless, management of ACM is complex due to the clinical heterogeneity of the disease, and optimal treatment protocols including risk stratification are still under development [5–8]. Studies on ACM often suffer from limitations secondary to the low prevalence and slow progression of the disease, i. e. many studies have insufficient statistical power and are restricted to retrospective follow-up since development of disease and (arrhythmic) endpoints is slow [9]. Additionally, the lack of uniform definitions complicates comparison of results among studies [10].

In order to overcome these limitations, we designed a national registry to include all Dutch ACM patients, first-degree relatives and/or carriers of ACM-associated pathogenic mutations. Observational clinical data are systematically collected (both retrospectively and prospectively) from first visit to last follow-up using uniform data collection instruments. In so doing, we aim to create a large national ACM patient cohort with a vast amount of uniformly collected high-quality data that is readily available for future research. The goals of this registry are to (1) increase the scientific knowledge base on disease mechanisms, genetics, and novel diagnostic and treatment strategies of ACM; and (2) use this platform to provide education for physicians and patients concerning ACM.

## Methods

### Design

The Netherlands ACM Registry is a national, multicentre observational cohort that is coordinated by the Netherlands Heart Institute (NHI, Utrecht, The Netherlands). The registry follows the Code of Conduct and the Use of Data in Heath Research and the national inclusion of patients is exempt from the Medical Research Involving Human Subjects Act (WMO) as per judgement of the Medical Ethics Committee (METC 18-126/C, Utrecht, The Netherlands). The ACM Registry is registered at the Netherlands Trial Registry, project 7097 (www.trialregister.nl).

### Objectives

The ACM Registry aims to (1) facilitate research on ACM disease mechanisms, genetics, diagnosis, prognosis, and treatment strategies; and (2) provide education for physicians and patients concerning ACM, e. g. through our website (www.acmregistry.nl) and patient conferences.

### Study population

Eligible for inclusion in the ACM Registry are: (1) index patients with a definite ACM diagnosis according to the diagnostic Task Force Criteria (TFC) [11] and in whom alternative diagnoses are excluded; (2) all first-degree relatives of ACM patients regardless of the index patient’s genetic testing results, which also includes relatives who are asymptomatic, who refuse genetic or cardiac testing, or those who are known to be mutation-negative (i. e. serve as control subjects); and (3) all carriers of pathogenic mutations in genes associated with ACM, regardless of their phenotype. After inclusion, a unique study ID is assigned to each registry enrolee by the NHI study coordinator to ensure the enrolee’s privacy. The study ID can be traced back to the enrolee only by the NHI coordinator and the local coordinator from the medical centre at which the enrolee is recruited. Currently, patients are recruited through all eight academic medical centres in the Netherlands.

### Data collection

Patient data are collected by researchers in the study centres using standardised data collection instruments hosted in REDCap (Research Electronic Data Capture, Vanderbilt University, Nashville, TN, USA) [12]. Supplementary Tab. 1 shows an overview of the collected clinical data with their definitions. In short, a comprehensive medical history is obtained, including demographics, symptoms, medication use, family history, molecular genetic analysis, pregnancy, and exercise history. Test results are ascertained at first presentation and at every follow-up visit, including laboratory values, (signal averaged) electrocardiograms, Holter recordings, exercise testing, electrophysiological studies, cardiac imaging, ventricular/coronary cine-angiograms, and cardiac tissue from biopsy or surgery. When available, raw data such as electrocardiogram tracings and de-identified images from cardiac imaging are stored through the Extensible Neuroimaging Archive Toolkit (XNAT, Washington University School of Medicine, St. Louis, MO, USA) software application for validation purposes and retrospective collection of newly identified relevant parameters. In addition, all interventions such as implantable cardioverter-defibrillator (ICD) placement and endocardial/epicardial ablations are recorded. As the registry design is observational, management and follow-up intervals remain at the discretion of the participant’s own cardiologist. Outcome data that are collected include (non-)sustained ventricular arrhythmias, ICD interventions, atrial arrhythmias, heart failure symptoms, hospitalisations, and (cardiac) death. The complete data dictionary and data collection instruments are available for download upon request.

### Data quality assurance

Data are acquired from routine clinical care in multiple academic centres that are all members of the Dutch Heritable Cardiomyopathy working group. Within this working group, clinical protocols regarding the diagnosis, genetic analysis and clinical care of cardiomyopathy patients are harmonised, which enhances the uniformity and quality of the data in this observational cohort. Uniform data collection is ensured by standardised data collection instruments built in REDCap accompanied by a detailed standard operating procedure document. Data entry fields are provided with entry instructions and are pre-programmed to accept values only within a possible range. The status of every data collection instrument is recorded: the default setting of ‘incomplete’ may be upgraded to ‘unverified’ when data are entered but not yet verified, and to ‘complete’ when data verification has been performed by an experienced researcher (rights are pre-specified in the researcher’s user account). All data access, entries and changes are recorded in a detailed audit trail by REDCap. The diagnostic criteria for ACM [11], dilated cardiomyopathy [13] and non-compaction cardiomyopathy [14, 15] are calculated by pre-programmed algorithms. Fulfilment of these criteria is thereby automatically determined in real time while entering the data to ensure accurate phenotyping.

### Data sharing and logistics

**Fig. 1:**
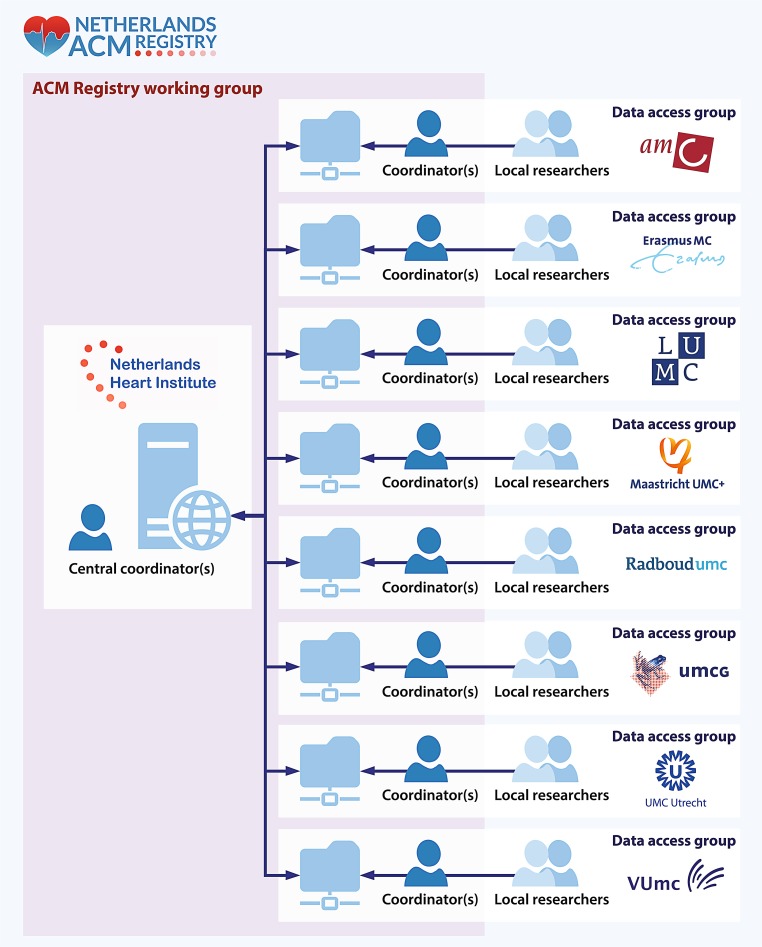
Graphic representation of the Netherlands ACM Registry: data access, logistics and sharing. The ACM Registry is hosted on a central server at the Netherlands Heart Institute. The database is divided in 8 data-access groups, managed by local coordinators of each participating centre. The central coordinators have access to the complete database for quality control and coordination of collaboration. The central coordinators together with the local coordinators form the ACM Registry working group

The REDCap database is hosted by the NHI. Security, data protection, and IT support are provided by the NHI Durrer Centre. Access to the ACM database is restricted to specific data access groups corresponding to the participating centres (Fig. 1) to ensure that researchers can access data only from patients known in their own centre. Only NHI research coordinators have access to the full database for quality assurance, database support, prevention of duplicate entries, and coordination of family linkage. Local coordinators are appointed in every centre to supervise data access and entry by local researchers. Together with the NHI coordinators, these local coordinators form the ACM Registry working group, which is tasked with discussing and coordinating data requests for multicentre studies. Prior to data release, the study protocol with research question, inclusion criteria, required data, and list of potential co-authors is approved by all collaborators to ensure scientific integrity. Researchers are free to use patient data within their data access group for local studies, provided that the ACM Registry and REDCap database are acknowledged.

## Results

As of 1 February 2018, the ACM Registry contains 850 individual patient records. Among these, 228 (27%) are ACM index patients and 622 (73%) are at-risk relatives, among whom 114 (18%) fulfil a definite ACM diagnosis. Pathogenic mutations are found in 69% of index patients (most commonly in plakophilin-2; 52%). An overview of the clinical characteristics is provided in Tab. 1.

**Table 1:** Clinical characteristics and available tests of 850 patients included in the Netherlands ACM Registry as of 1 February 2018

Patient characteristics	All	Index patients	Family members
Number	850 (100.0%)	228 (26.8%)	622 (73.2%)
Age at presentation (years)	38 [24–50]	39 [27–46]	38 [21–52]
Male sex	443 (52.1%)	161 (70.6%)	282 (45.3%)
ACM diagnosis^a^			
– definite	342 (40.2%)	228 (100%)	114 (18.3%)
– borderline	90 (10.6%)	n. a.	90 (14.5%)
Genetic testing performed	702 (82.6%)	226 (99.1%)	476 (76.5%)
Pathogenic mutation	458 (65.2%)	157 (69.5%)	301 (63.2%)
– *PKP2*	361 (51.4%)	118 (52.2%)	243 (51.1%)
– *DSP*	7 (1.0%)	4 (1.8%)	3 (0.6%)
– *JUP*	0 (0.0%)	0 (0.0%)	0 (0.0%)
– *DSG2*	14 (2.0%)	6 (2.7%)	8 (1.7%)
– *DSC2*	11 (1.6%)	5 (2.2%)	6 (1.3%)
– *PLN*	63 (9.0%)	24 (10.6%)	39 (8.2%)
– other	12 (1.7%)	5 (2.2%)	7 (1.5%)
– multiple^b^	9 (1.3%)	4 (1.8%)	5 (1.1%)
*Test results available (≥1)*			
ECG	674 (79.3%)	215 (94.3%)	459 (73.8%)
SAECG	88 (10.4%)	50 (21.9%)	38 (6.1%)
ETT	397 (46.7%)	166 (72.8%)	231 (37.1%)
Holter monitoring	495 (58.2%)	166 (72.8%)	329 (52.9%)
Imaging	576 (67.8%)	210 (92.1%)	366 (58.8%)
– echo	550 (64.7%)	206 (90.4%)	344 (55.3%)
– MRI	389 (45.8%)	170 (74.6%)	219 (35.2%)
– angiogram	193 (22.7%)	150 (65.8%)	43 (6.9%)
EPS	169 (19.9%)	133 (58.3%)	36 (5.8%)
Tissue biopsy	115 (13.5%)	89 (39.0%)	26 (4.2%)
*Follow-up*			
Follow-up available	384 (45.2%)	210 (92.1%)	174 (28%)
– duration (years)	9.5 [4.6–16.2]	12.2 [5.1–20.0]	7.6 [3.3–12.1]
ICD implanted	235 (27.6%)	165 (72.4%)	70 (11.3%)
Sustained VA	196 (23.1%)	163 (71.5%)	33 (5.3%)
Heart transplantation	7 (0.8%)	5 (2.2%)	2 (0.3%)
Death	53 (6.2%)	36 (15.8%)	17 (2.7%)

*ACM* arrhythmogenic cardiomyopathy, *DSC2* desmocollin-2, *DSG2* desmoglein-2, *DSP* desmoplakin, *ECG* electrocardiogram, *EPS* electrophysiologic study, *ETT* exercise treadmill test, *ICD* implantable cardioverter-defibrillator, *JUP* junction plakoglobin, *MRI* magnetic resonance imaging, *PKP2* plakophilin-2, *PLN* phospholamban, *SAECG* signal-averaged electrocardiogram, *TFC* task force criteria, *VA* ventricular arrhythmia

^a^Definite ACM is defined as modified TFC score ≥4; borderline ACM is defined as modified TFC score 3

^b^Digenic or compound heterozygous

Follow-up information is currently available for 384 (45%) patients, among whom 210 (92%) are index patients and 174 (28%) relatives. Median follow-up is 9.5 years (interquartile range 4.6–16.2). The available clinical tests are outlined in Tab. 1. At least one electrocardiogram is available for almost all index patients (*n* = 215, 94%) and most relatives (*n* = 459, 74%), while Holter monitoring is available in the majority of both groups (*n* = 166, 73% and *n* = 329, 53%, respectively). An electrophysiological study is available in 133 (58%) index patients and 36 (6%) family members. Almost all index patients (*n* = 210, 92%) and most family members (*n* = 366, 59%) underwent at least one modality of cardiac imaging, with echocardiography being the most common (*n* = 206, 90% and *n* = 344, 55%, respectively), followed by cardiac magnetic resonance (*n* = 170, 75% and *n* = 219, 35%, respectively), and angiography (*n* = 150, 66% and *n* = 43, 7%, respectively).

## Discussion

Clinical research on ACM is often limited by (1) small patient numbers; (2) retrospective study designs; and (3) inconsistent data definitions, leading to inability to compare results across studies [10]. To overcome these limitations, collaboration and sharing of expertise is paramount.

In the past, collaborative ACM research using multinational transatlantic databases has provided strong evidence on several clinically relevant problems including diagnosis, genotype-phenotype correlations and family screening [11, 16–18]. While Dutch ACM patients have previously been enrolled in these studies, data collection was largely cross-sectional and hypothesis-driven, hence only applicable to one specific study. This, as well as the introduction of new data collection guidelines (e. g. standardised case record forms and audit trails), demanded a new platform. With the Netherlands ACM Registry, we designed a platform to create and maintain a large observational longitudinal patient cohort that continues and expands our prior database to a user-friendly and sustainable ACM Registry.

In the Netherlands ACM Registry, we use standardised protocols to ensure uniform, high-quality data. All these data are readily available to facilitate collaborative ACM research. A wide range of demographic and clinical data are collected including disease phenotype, genotype, treatment, and outcomes at multiple time points, enabling both cross-sectional and longitudinal studies in a hypothesis-generating approach. Data validation occurs through several automated validation processes (e. g. real-time calculation of diagnostic TFC) which undergo an additional manual check by experts (e. g. electrocardiogram over-read by trained electrophysiologists). Final ACM diagnosis is manually confirmed by experts. The phenotype algorithms aid long-term sustainability of the database, as they can easily be altered if the diagnostic guidelines are modified. In addition, data validity and sustainability are assured by storing raw data such as de-identified cardiac magnetic resonance images, which can be re-evaluated if new insights are gained.

One limitation of our registry is the observational nature, in which we do not impose standard clinical evaluation intervals or interfere with diagnostic and/or treatment strategies. This may introduce centre-specific differences, which should be accounted for in every study separately depending on the research question. Furthermore, our registry is phenotype-based, meaning that inclusion of patients and relatives is restricted to families in which at least one relative has a definite diagnosis of ACM [11] and ACM-related mutation carriers. Although we consider this to be a strength to minimise distortion of results by inclusion of non-ACM patients, this also introduces limitations: at the present time, our registry cannot be used to study the differentiation of ACM with disease-mimicking entities.

### Future perspectives

We aim to improve this registry continuously. Future perspectives include the expansion of the population to borderline/possible ACM patients. We also plan to collaborate with existing biobanks for cardiac tissue, DNA and plasma to facilitate additional research on disease penetrance and the pathophysiological mechanisms of ACM. The Netherlands ACM Registry aims to stimulate existing and future (inter-)national collaboration and transparency in ACM research, not only among researchers but also between researchers and patients. In the future, we intend to use this registry as a tool to enable physician and patient education by means of patient conferences as well as to provide interested readers with the possibility to receive updates on current and future research in newsletters.

## Conclusion

The Netherlands ACM Registry is a national observational cohort of ACM patients and at-risk relatives. Data collection is performed both prospectively and retrospectively using a secure online platform that includes demographic, genetic, and clinical characteristics at multiple time points, enabling both cross-sectional and longitudinal research. By using uniform variable definitions and automatic data verification, a user-friendly and sustainable platform is generated. The final aim of this registry is to (1) increase the scientific knowledge base of ACM by strong national collaboration, as well as facilitating potential international collaborations; and (2) provide education for physicians and patients concerning ACM.

## Caption Electronic Supplementary Material

Supplementary Table 1 ACM Registry data dictionary
