# Supplementary material for: The Netherlands Arrhythmogenic Cardiomyopathy Registry: design and status update
Source: Neth Heart J. 2019 Apr 17;27(10):480–6. doi: 10.1007/s12471-019-1270-1 (PMC6773794; doi:10.1007/s12471-019-1270-1)
Supplement: Supplementary file 1 — Supplementary Table 1 ACM Registry data dictionary [file 12471_2019_1270_MOESM1_ESM.docx]

**Supplementary Table 1.** ACM Registry data dictionary

| **Demographics** | | |
| --- | --- | --- |
| Country | Free text | *Country of residence* |
| Centre | Free text | *Centre of enrolment* |
| Year of birth | YYYY | *Year in which the patient was born* |
| Sex | - Female - Male | *Sex of the patient* |
| Pedigree | - Proband - Family member | *Proband (‘index patient’) is defined as the first affected family member seeking medical attention for ACM-related complaints in whom the diagnosis was confirmed (i.e. an individual ascertained independently of family history).* |
| Ethnicity | - Caucasian - Asian - African (-American) - Hispanic - Mixed | *Ethnicity of the patient* |
|  |  |  |
| **Presentation & symptoms** | | |
| Date of presentation | DD/MM/YYYY |  |
| Type of presentation | - Sudden cardiac death - Symptomatic and living - Resuscitated sudden cardiac arrest - Abnormal test - Family history | *Symptomatic is defined as having symptoms attributed to ACM (syncope, pre-syncope, palpitations, chest pain).* |
| Ventricular arrhythmia | Duration; morphology; cycle length. |  |
| Symptoms | - Cardiac syncope - Presyncope - Palpitations - Dyspnoea - Chest pain | *Cardiac syncope is defined as transient loss of consciousness and postural tone with spontaneous recovery with a likely arrhythmic mechanism.* |
| NYHA class | I - IV | *Functional classification as defined by the New York Heart Association.* |
| Comorbidities | - Hypertension - Diabetes Mellitus - Dyslipidaemia - Myocardial infarction - Peripheral vascular disease - Cerebrovascular accident /   Transient ischemic attack   - COPD - Sarcoidosis |  |
|  |  |  |
| **Family history** | | |
| Date of ascertainment of family history | DD/MM/YYYY |  |
| Degree of relatedness to the index patient | - First degree - Second degree | *First degree is defined as family members with 50% relatedness (i.e. parents, siblings and children). Second degree is defined as family members with 25% relatedness (i.e. grandparents, grandchildren, aunts, uncles, nephews, nieces, etc.)* |
| Family history of heart disease | - ACM/ARVC   - Task force diagnosis   - Autopsy diagnosis   - Assumed diagnosis - DCM - HCM - Other (specify) | *Assumed diagnosis is defined as diagnosis not confirmed by Task Force criteria or autopsy* |
|  |  |  |
| **Genetics** |  |  |
| Date of genetic testing | DD/MM/YYYY |  |
| Type of analysis | - Sanger sequencing - Gene panel(s) - CNV detection software - Multiplex ligation-dependent probe amplification |  |
| Gene tested | - Plakophilin-2 *(PKP2)* - Desmoplakin *(DSP)* - Junctional plakoglobin (*JUP*) - Desmoglein-2 (*DSG2*) - Desmocolin-2 (*DSC2*) - Transmembrane protein 43 (*TMEM43*) - Transforming growth factor β3 (*TGFβ3*) - Phospholamban (*PLN*) - Titin (*TTN*) - Desmin (*DES*) - Lamin A/C (*LMNA*) - Ryanodine receptor 2 (*RYR2*) - Voltage-gated sodium channel α-subunit 5 (*SCN5A*) - N-cadherin (*CDH2*) - Catenin α3 (*CTNNA3*) - Other variants found (specify) |  |
| If variant found: | - Reference sequence number - Nucleotide variant - Amino acid change - Homozygous, heterozygous, compound heterozygous - Pathogenicity classification | *Pathogenicity classification as per ACMG guidelines. Nonsense, frameshift, splice site mutations and exon deletions are considered proven pathogenic unless previously identified as polymorphism. Missense mutations are considered pathogenic when 1) minor allele frequency in Exome Sequencing Project (ESP) was ≤0.05%, (NHLBI 6500 Exome data sets; EVS; http://evs.gs.washington.edu/EVS/) and 2) in silico prediction programs predicted the variant to affect protein function by score <0.02 (SIFT) and >0.900 (Polyphen2).* |

|  |  |  |
| --- | --- | --- |
| **Exercise history** | | |
| Endurance athlete | Yes/no | *Defined as Bethesda class C (High dynamic component >70% max O_2_)* |
| Types of sport | Free text | *E.g. Soccer, tennis, basketball, etc.* |
| Activity level | Low  Moderate  High |  |
| Competitive athlete | Yes/no |  |
|  |  |  |
| **Medication** | | |
| Date of medication log | DD/MM/YYYY |  |
| Beta-blockers | - Yes (specify name + dose) - No |  |
| Anti-arrhythmic drugs | - Class 1A (specify name + dose) - Class 1B (specify name + dose) - Class 1C (specify name + dose) - Class 3 (specify name + dose) - Class 4 (specify name + dose) | *NB Sotalol classified as class 3 anti-arrhythmic drug irrespective of dose* |
| Diuretics | - Yes (specify name + dose) - No |  |
| ACE-inhibitors / ARBs | - Yes (specify name + dose) - No |  |
|  |  |  |
| **Electrocardiogram** | | |
| Date of ECG | DD/MM/YYYY |  |
| Upload anonymized ECG | Upload button | *Anonymization facilitated by automatic redaction tool* |
| Medication used during recording | Free text |  |
| Rhythm | - Sinus rhythm - Atrial pacing - (Atrial-)ventricular pacing - Other (free text) |  |
| Heart rate frequency | (bpm) | *Allowed range 10 - 400* |
| QRS duration | (ms) | *Maximal QRS duration on ECG Allowed range 20-400* |
| R-axis | (degrees) | *Allowed range -90 - 270* |
| PQ duration | (ms) | *Allowed range 20-400* |
| QT interval | (ms) | *Allowed range 100-700* |
| Bundle branch block | - Complete RBBB - Atypical complete RBBB - Complete LBBB - Non-specific intraventricular conduction delay | *Criteria for typical right and left bundle branch block criteria as per WHO criteria* |
| Terminal activation duration | - >55 ms (Yes/No) - Absolute duration (ms) | *TAD is defined as the longest duration in V1-3, from the nadir of the S wave to the end of all depolarization deflections including R’, in the absence of typical complete right bundle-branch block* |
| Epsilon wave | - Yes - No | *Distinct waves of small amplitude within the ST segment in the right precordial leads (V1-3) which are distinct from the QRS complex.* |
| T-wave inversion | - V1 - V2 - V3 - V4 - V5 - V6 - II - III - aVF | *Inverted T-waves are recorded per lead.*  *T-waves are considered inverted if amplitude ≥ 1 mV.* |
| Presence of PVC(s) | Yes/No; number; morphology. |  |
| Low QRS voltage | - Leads I, II and III all <0.5 mV - Leads I+II+III <1.5 mV - Leads V1-6 all <1.0 mV - Other (free text) |  |
|  |  |  |
| **Signal-averaged Electrocardiogram (SAECG)** | | |
| Date of SAECG | DD/MM/YYYY |  |
| Upload anonymized SAECG | Upload button | *Anonymization facilitated by automatic redaction tool* |
| Filtered QRS duration | (ms) | *Allowed range 60-300* |
| Duration of terminal QRS <40mV | (ms) | *Allowed range 0-100* |
| Root mean square voltage of terminal 40ms | (mV) | *Allowed range 0-100* |
|  |  |  |
| **Holter monitoring** | | |
| Date of Holter monitor | DD/MM/YYYY |  |
| Upload copy of Holter monitor report | Upload button | *Anonymization facilitated by automatic redaction tool* |
| Use of cardiac medication during recording | Free text |  |
| Monitoring time | hours | *Allowed range 12-50* |
| Total PVC count | number | *Allowed range 0-200000* |
| Ventricular arrhythmia | Duration; morphology; cycle length. |  |
|  |  |  |
| **Exercise tolerance test** | | |
| Date of exercise tolerance test | DD/MM/YYYY |  |
| Upload anonymized exercise tolerance test | Upload button | *Anonymization facilitated by automatic redaction tool* |
| Cardiac medication during test | Free text |  |
| Baseline blood pressure | (mmHg) | *Allowed range 40-250 / 20-180* |
| Maximum blood pressure | (mmHg) | *Allowed range 40-250 / 20-180* |
| Ventricular tachycardia | Duration; morphology; cycle length. |  |
| PVC(s) | Presence; morphology. |  |
| Other arrhythmia(s) | Free text |  |
|  |  |  |
| **Electrophysiology study (EPS)** | | |
| Date of EPS | DD/MM/YYYY |  |
| Upload copy of EPS report | Upload button | *Anonymization facilitated by automatic redaction tool* |
| Cardiac medication during EPS | Free text |  |
| Ventricular arrhythmia induced at stimulation | Duration; morphology; cycle length. |  |
| Induction method | - Programmed ventricular stimulation - Isoproterenol infusion |  |
| Late potentials | - Yes - No | *Considered positive if potentials are recorded on intracardiac electrogram after the end of the QRS-complex on the surface ECG.* |
| Ablation performed | - Yes - No   If yes:   - Endocardial location(s) - Epicardial location(s) - Both endo- and epicardial location(s) |  |
|  |  |  |
| **Magnetic resonance imaging (MRI)** | | |
| Date of MRI | DD/MM/YYYY |  |
| Upload copy of MRI report | Upload button | *Anonymization facilitated by automatic redaction tool* |
| Body surface area at time of test | (m^2^) | *As calculated by DuBois formula (height^0.725)*(length^0.425)*0.007184* |
| Global RV dilatation | - Mild - Moderate - Severe | *Qualitative assessment*  *Mild: RV diameter < LV diameter*  *Moderate: RV diameter = LV diameter*  *Severe: RV diameter > LV diameter* |
| Global RV dysfunction | - Mild - Moderate - Severe | *Qualitative assessment* |
| Regional RV wall motion abnormalities | - Hypokinesia (specify region) - Akinesia (specify region) - Dyskinesia (specify region) - Aneurysm (specify region) | *Qualitative assessment* |
| RV measurements | - End-diastolic volume (mL) - End-systolic volume (mL) - Ejection fraction (%) |  |
| Global LV dilatation | - Mild - Moderate - Severe | *Qualitative assessment* |
| Global LV dysfunction | - Mild - Moderate - Severe | *Qualitative assessment* |
| Regional LV wall motion abnormalities | - Hypokinesia (specify region) - Akinesia (specify region) - Dyskinesia (specify region) - Aneurysm (specify region) | *Qualitative assessment* |
| LV measurements | - End-diastolic volume (mL) - End-systolic volume (mL) - Ejection fraction (%) |  |
| Dyssynchronous movement | - Dyssynchronous contraction - Dyssynchronous relaxation | *Qualitative assessment* |
| Fatty infiltration | - Yes (specify region) - No | *Qualitative assessment* |
| Late gadolinium enhancement | - Yes (specify region) - No | *Qualitative assessment* |
| Atrial dilatation | - Left - Right - Both | *Qualitative assessment* |
| Abnormal feature tracking | - Yes (specify region) - No |  |
| T1 mapping performed | - Yes - No |  |
| Signs of non-compaction | - RV - LV - Both |  |
|  |  |  |
| **Echocardiogram** | | |
| Date of echocardiogram | DD/MM/YYYY |  |
| Upload copy of echocardiogram report | Upload button | *Anonymization facilitated by automatic redaction tool* |
| Body surface area at time of test | (m^2^) | *Calculated by DuBois formula (height^0.725)*(length^0.425)*0.007184* |
| Global RV dilatation | - Mild - Moderate - Severe | *Qualitative assessment*  *Mild: RV diameter < LV diameter*  *Moderate: RV diameter = LV diameter*  *Severe: RV diameter > LV diameter* |
| Global RV dysfunction | - Mild - Moderate - Severe | *Qualitative assessment of RV function* |
| Regional RV wall motion abnormalities | - Hypokinesia (specify region) - Akinesia (specify region) - Dyskinesia (specify region) - Aneurysm (specify region) | *Qualitative assessment* |
| RV measurements | - Fractional area change (%) - Tricuspid annular plane systolic excursion (mm) - Outflow tract (PLAX)(mm) - Outflow tract (PSAX)(mm) |  |
| Global LV dilatation | - Mild - Moderate - Severe | *Qualitative assessment* |
| Global LV dysfunction | - Mild - Moderate - Severe | *Qualitative assessment* |
| LV measurements | - Ejection fraction (%) - Fractional shortening (%) - End-diastolic volume (mL) |  |
| Abnormal deformation imaging | - Yes (specify region) - No |  |
| Atrial dilatation | - Left - Right - Both |  |
| Signs of non-compaction | - RV - LV - Both |  |
|  |  |  |
| **Angiogram** | | |
| Date of angiogram | DD/MM/YYYY |  |
| Upload copy of angiogram report | Upload button | *Anonymization facilitated by automatic redaction tool* |
| Global RV dilatation | - Yes - No | *Qualitative assessment* |
| Regional RV regional wall motion abnormalities | - Akinesia, dyskinesia or aneurysm (specify region) - Hypokinesia (specify region) - No | *Qualitative assessment* |
| Global LV dilatation | - Yes - No | *Qualitative assessment* |
| Regional LV regional wall motion abnormalities | - Akinesia, dyskinesia or aneurysm (specify region) - Hypokinesia (specify region) - No | *Qualitative assessment* |
| Coronary artery disease | - Yes - No | *Defined as >=75% stenosis in a major epicardial coronary artery* |
|  |  |  |
| **Tissue histology** | | |
| Date that specimen is obtained | DD/MM/YYYY |  |
| Upload copy of pathology report | Upload button | *Anonymization facilitated by automatic redaction tool* |
| Source of tissue | - Biopsy - Autopsy - Transplantation - Other |  |
| Fulfilment of Arrhythmogenic Cardiomyopathy diagnostic criteria | - Major - Minor - None | *As defined by the 2010 TFC: Major if < 60% residual myocytes by morphometric analysis (or < 50% if estimated), with fibrous replacement of the*  *RV free wall myocardium >=1 sample, with or without fatty replacement of tissue on*  *endomyocardial biopsy; Minor if 60% to 75% residual myocytes by morphometric analysis (or 50% to 65% if estimated), with fibrous replacement of the RV free wall myocardium in >=1 sample, with or without fatty replacement of tissue on endomyocardial biopsy.* |
|  |  |  |
| **Device implantation** | | |
| Date of implantation | DD/MM/YYYY |  |
| Copy of device readouts / settings summary | Upload button | *Anonymization facilitated by automatic redaction tool* |
| Type of device | - ICD: Single chamber - ICD: Dual chamber or CRT-D - S-ICD - Pacemaker: single chamber - Pacemaker: dual chamber - Pacemaker: leadless - Other (specify) | *RV lead only = single chamber. RV and RA lead = dual chamber. RA, RV and LV lead = CRT-(D), subcutaneous ICD = S-ICD.* |
| Type of implantation | - New implantation - Generator replacement - Lead revision - Other (specify) |  |
| Defibrillator indication | - Primary prevention - Secondary prevention | *Secondary prevention if previously documented sustained VT/VF. If the ICD indication is based on syncope without registration of a ventricular arrhythmia it is regarded as primary prevention.* |
| Defibrillator settings | - Rate cut-off for anti-tachycardia pacing or shock - Rate cut-off for monitoring window | *For anti-tachycardia pacing or shock, note lowest rate at which device provides therapy.*  *For rate cut-off specify cycle length~~s~~ in ms.* |
|  |  |  |
| **Arrhythmic event / ICD intervention** | | |
| Date of arrhythmic event | DD/MM/YYYY |  |
| Upload copy of event registration | Upload button | *Anonymization facilitated by automatic redaction tool* |
| Documentation type | - ECG recording - ICD recording - Other (specify) |  |
| Cardiac medication at time of event | Free text |  |
| Event type | - Spontaneous VT/VF - Appropriate ICD intervention (anti-tachycardia pacing or shock) - VT-storm / electrical storm - Aborted SCD | *VT storm is defined by >2 sustained arrhythmias (or appropriate ICD interventions) within 24h.* |
| Ventricular tachycardia or ICD intervention | Duration; morphology; cycle length | *Ventricular tachycardia is considered sustained if lasting 30 seconds or more, or less than 30 seconds when terminated electrically or pharmacologically* |
| Type of ICD intervention | - Anti-tachy-pacing (ATP) - Shock |  |
| Circumstances event | - Routine activity - Rest - Sleep - Exercise |  |
|  |  |  |
| **Inappropriate ICD intervention** | | |
| Date of inappropriate ICD intervention | DD/MM/YYYY |  |
| Upload copy of event registration | Upload button | *Anonymization facilitated by automatic redaction tool* |
| Type of intervention | - Anti-tachycardia pacing (ATP) - Shock |  |
| Cause | - Atrial arrhythmia - Sinus tachycardia - Lead or device malfunction - Other (specify) |  |
|  |  |  |
| **Atrial arrhythmia** | | |
| Date of atrial arrhythmia | DD/MM/YYYY |  |
| Upload copy of event registration | Upload button | *Anonymization facilitated by automatic redaction tool* |
| Type | - Atrial fibrillation - Atrial flutter - Other (specify) |  |
| Documentation type | - ECG recording - ICD recording - Holter recording - Exercise test - Other (specify) |  |
|  |  |  |
| **Pregnancy** | | |
| Number of pregnancies | Number; date of delivery |  |
| Cardiac complications associated with pregnancy | - Yes (specify) - No - Unknown | *E.g. symptoms of heart failure or arrhythmia in the mother, obstetric complications in the child* |
|  |  |  |
| **Heart failure** | | |
| Date of onset heart failure | DD/MM/YYYY | *Defined as a clinical syndrome with symptoms as dyspnoea, fatigue, limited exercise tolerance, and/or fluid retention caused by a structural and/or functional cardiac abnormality. (Definitions from: ACCF/AHA 2013, ESC 2016).* |
| Date of first hospitalization for heart failure | DD/MM/YYYY |  |
|  |  |  |
| **Heart transplantation / ventricular assist device** | | |
| Date of transplantation / VAD implantation | DD/MM/YYYY |  |
| Type | - Heart transplantation - LVAD - RVAD - BiVAD - Other |  |
| Indication | - Incessant ventricular arrhythmia - RV failure - LV failure - Biventricular failure - Other (specify) |  |
|  |  |  |
| **Death** | | |
| Date of death | DD/MM/YYYY |  |
| Cause | - Cardiovascular   - Sudden cardiac death   - Heart failure / shock   - Other (specify) - Non-cardiovascular (specify) |  |

|  |  |  |
| --- | --- | --- |
| **Diagnostic criteria** | | |
| Fulfilment of criteria for ARVC | Total TFC score; automatically calculated by software based on previous entry sheets | *Definite diagnosis ≥ 4 TFC criteria*  *Borderline diagnosis: 3 TFC criteria*  *Possible diagnosis: 2 TFC criteria* |
| Fulfilment of criteria for ARVC; by category | - Family history / genetics - Depolarization - Repolarization - Arrhythmia - Structural (imaging) - Tissue   Automatically calculated by software based on previous entry sheets |  |
| Fulfilment of criteria for DCM | Automatically calculated by software based on previous entry sheets | *If LVEDD>117% of the predicted value, and a reduced LV function (EF< 45% or FS< 35%)* |
| Coronary artery disease | Automatically calculated by software based on previous entry sheets | *If CTA calcium score >10 and/or CAG stenosis >= 75%* |
| Fulfilment of criteria for non-compaction | Automatically calculated by software based on previous entry sheets | *If non-compacted / compacted layer ratio on MRI is >2.3, or the end systolic non-compacted / compacted layer ratio in echocardiogram is >2.0* |
